# Humoral immunity to phlebovirus infection

**DOI:** 10.1111/nyas.15080

**Published:** 2023-11-07

**Authors:** Katie J. Doores

**Affiliations:** ^1^ Department of Infectious Diseases, King's College London Guy's Hospital London UK

**Keywords:** neutralizing antibody, neutralizing epitope, phenuiviridae, phlebovirus, vaccine, zoonosis

## Abstract

Phleboviruses are zoonotic pathogens found in parts of Africa, Asia, Europe, and North America and cause disease symptoms ranging from self‐limiting febrile illness to severe disease, including hemorrhagic diathesis, encephalitis, and ocular pathologies. There are currently no approved preventative vaccines against phlebovirus infection or antivirals for the treatment of the disease. Here, we discuss the roles of neutralizing antibodies in phlebovirus infection, the antigenic targets present on the mature polyproteins Gn and Gc, progress in vaccine development, and the prospects of identifying conserved neutralizing epitopes across multiple phleboviruses. Further research in this area will pave the way for the rational design of pan‐phlebovirus vaccines that will protect against both known phleboviruses but also newly emerging phleboviruses that may have pandemic potential.

## Graphical abstract

Phleboviruses are global zoonotic pathogens that can cause severe disease when transmitted to humans. Here we discuss how studying antibody responses during natural infection and the identification of neutralizing epitopes on the mature polyproteins Gn and Gc are informing the development of phlebovirus vaccines.

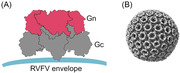

## BIOMEDICAL BURDEN OF PHLEBOVIRUSES

Phleboviruses are a genetically diverse group of viruses from the family *Phenuiviridae*, order *Bunyavirales*. A well‐known phlebovirus member is Rift Valley Fever Virus (RVFV), an arbovirus that is the causative agent of Rift Valley Fever (RVF). Endemic to Africa^1^ and more recently the Arabian Peninsula,^2^ it can cause recurrent epidemics, with human disease ranging from mild self‐limiting febrile illness to severe disease, including hemorrhagic diatheses, encephalitis, and ocular pathologies.^3^, ^4^ Ruminants are the major reservoirs of RVFV, and infection in livestock leads to high rates of neonatal mortality, resulting in major losses to the livestock industry in endemic areas.^5^ RVFV is transmitted to humans through bites of infected mosquitos or through contact with the blood or organs of infected animals.^6^

There are 10 other reported species within the phlebovirus family, of which some have been reported in repeated zoonotic events.^7^ Toscana virus (TOSV) is a phlebovirus isolated from two species of sandfly in Italy.^8^ Infections mostly occur in Mediterranean countries in the summer months^9^ and can cause acute neurological diseases. Heartland virus (HRTV) is the first pathogenic phlebovirus observed in the United States^10^, ^11^ and is most closely related to severe fever with thrombocytopenia syndrome virus (SFTSV). SFTSV, which was first reported in China in 2007,^12^ was originally classified as a phlebovirus but was recently reclassified in the genus *Bandavirus* of the *Phenuiviridae* family, order *Bunyavirales* ^13^ and officially named Dabie bandavirus. Due to the previous classification of SFTSV as a phlebovirus and the literature comparing SFTSV and RVFV structures and antibody responses,^14^, ^15^, ^16^ SFTSV has been included in this review. SFTSV is characterized by a sudden onset fever, respiratory or gastrointestinal symptoms, and progression to hemorrhage and multiorgan failure,^17^ and it has an average case fatality rate of 12%.^18^ Transmission of SFTSV is predominantly associated with arthropod bites but person‐to‐person transmission through blood contact has also been reported.^18^, ^19^ High SFTSV seroprevalence in domestic animals has been reported in endemic regions, demonstrating a number of different vertebrate hosts.^20^, ^21^

Phleboviruses are a significant zoonotic and pandemic threat to both human and animal populations due to the global presence of both the hosts (including livestock and domestic animals) and the arthropod vectors (including mosquito, tick, and sandfly). Indeed, the World Health Organization has reported nine outbreaks of RVFV between 2000 and 2016, infecting over 4600 people and leading to 957 deaths.^6^ However, there are currently no approved vaccines for the prevention of phlebovirus infection or antivirals for the treatment of the disease, although several vaccine approaches^22^, ^23^, ^24^, ^25^ and antivirals^26^, ^27^ are currently being explored.

## PHLEBOVIRUS STRUCTURE

**FIGURE 1:**
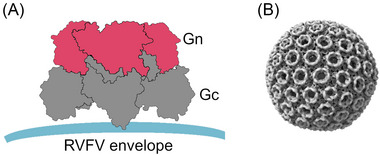
RVFV Gn‐Gc assembly. (A) Schematic showing the arrangement of RVFV Gn (pink) and Gc (dark gray) on the virion surface. (B) Gn–Gc complex assembly of RVFV (image created in ChimeraX using EMB‐4197).^31^

Phleboviruses are enveloped single‐stranded negative‐sense RNA viruses, and their genomes are divided into three segments: S, M, and L. The S segment encodes the nucleoprotein and a nonstructural protein (NSs), the M segment encodes the structural glycoproteins, and the L segment encodes the RNA‐dependent RNA polymerase. The M segment encodes a polyprotein that is cleaved to form two mature polyproteins, Gn and Gc, which associate to form a heterodimer (Figure 1A), which is then arranged into pentamers and hexamers that encapsulate the virion (Figure 1B).^28^, ^29^, ^30^, ^31^ Gn and Gc are essential for viral attachment and host cell entry. In the case of RVFV, host‐cell entry begins with the interaction between the high‐mannose glycans on Gn and the C‐type lectin DC‐SIGN.^32^ This interaction initiates caveolae‐mediated endocytosis.^33^ Gc harbors a class II fusion protein and facilitates membrane fusion utilizing a pH‐dependent mechanism in the acidic environment of an endosomal compartment.^31^

**FIGURE 2:**
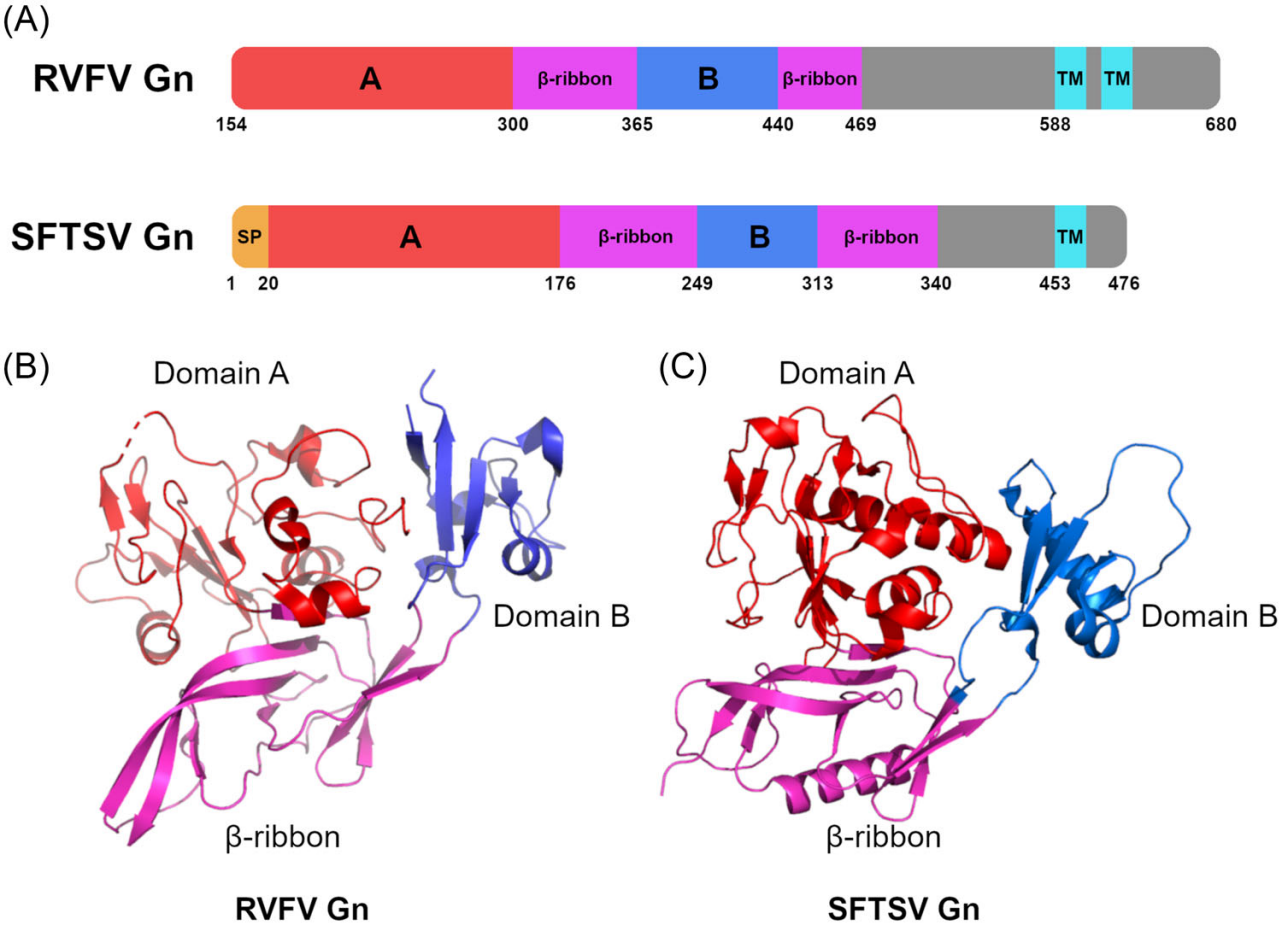
Phlebovirus Gn structure. (A) Schematic showing the domain organization for RVFV and SFTSV Gn. Structurally identified domains are colored (domain A: red; domain B: blue;  β‐ribbon domain: pink). (B) Structure of RVFV Gn (PDB: 6F8P^31^), with structural domains colored as in panel A. (C) Structure of SFTSV Gn (PDB: 5Y10^16^).

**FIGURE 3:**
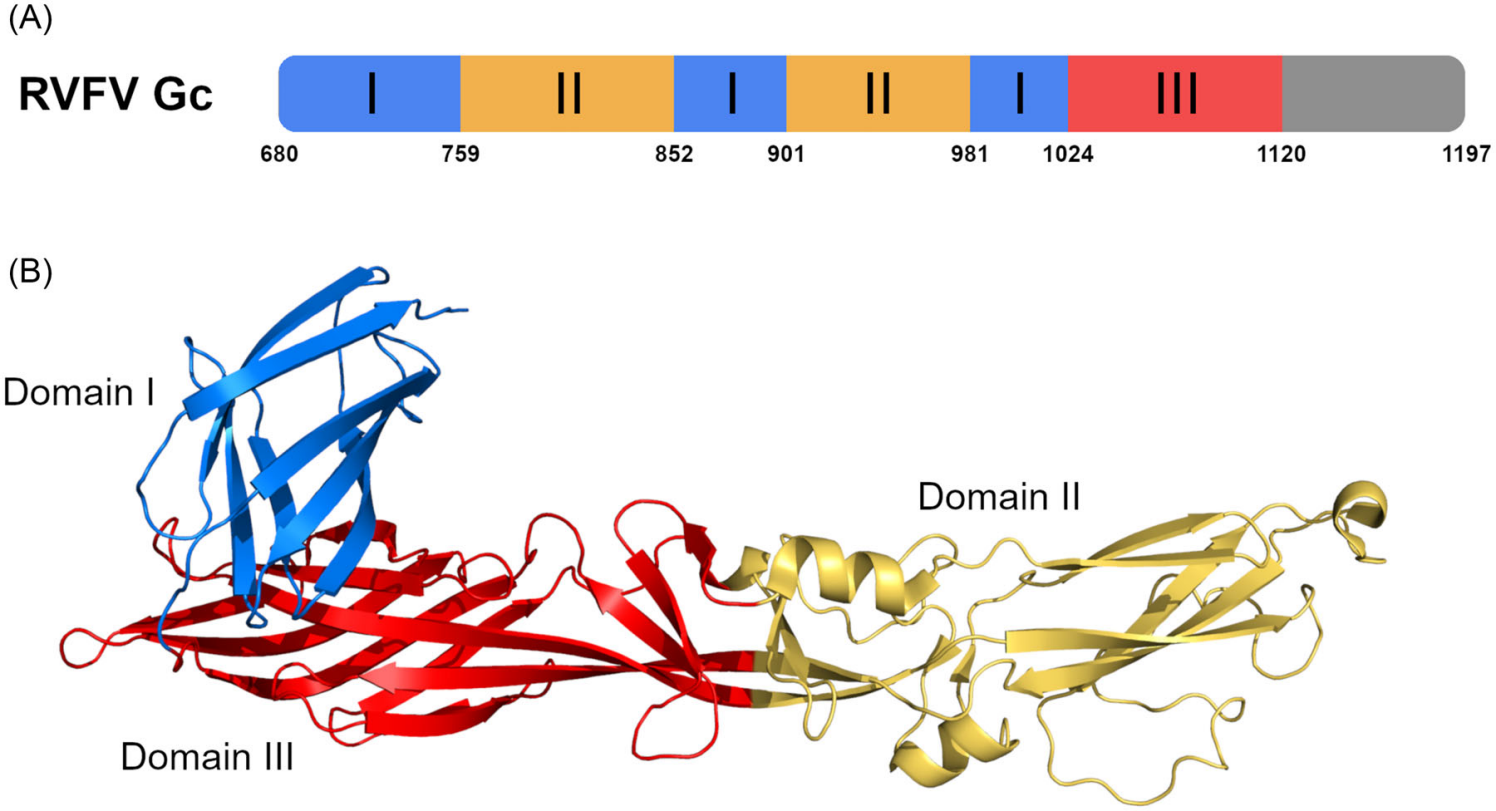
Phlebovirus Gc structure. (A) Domain organization of RVFV Gc. Structurally identified domains are colored (domain I: blue; domain II: yellow; domain III: red). (B) Structure of RVFV Gc (PBD: 4HJ1^34^), with structural domains colored as in panel A.

Crystal structures of the Gn of RVFV^14^, ^15^, ^31^ and SFTSV^16^ reveal a three‐domain architecture consisting of (i) domain A, an α‐helical/β‐stranded N‐terminal domain; (ii) a β‐ribbon domain; and (iii) domain B (Figure 2A). However, although the Gn molecules from RVFV and SFTSV display the same overall fold (Figure 2B,C), there are differences in the arrangement of subdomains, demonstrating the structural variation between *Phenuiviridae* family members.^16^ In contrast to Gn, the Gc structure is relatively conserved between RVFV,^34^ SFTSV,^35^ and HRTV.^36^ Gc has three domains (termed I–III) that are composed of β‐sheets (Figure 3A,B). Domain II contains the fusion loops. Localization of the crystalized fragments of RVFV Gn and Gc in purified RVFV cryo‐EM‐based single particle reconstructions shows that the N‐terminal domain of Gn is membrane‐distal to the viral envelope and shields both Gc and its hydrophobic fusion loop, preventing premature fusion.^31^ Further structural studies comparing the higher‐order ultrastructures of SFTSV and HRTV are required to determine whether this arrangement is conserved across the phlebovirus family.^37^

## HUMORAL IMMUNE RESPONSE TO PHLEBOVIRUS INFECTION

Gn and Gc are the key targets for neutralizing antibodies elicited following infection and, therefore, the main antigens of interest for vaccine development. Neutralizing antibodies have been identified following RVFV infection in livestock,^1^ nonhuman primates,^38^ and humans.^39^ The protective capacity of neutralizing antibodies has been demonstrated through passive transfer of convalescent sera to susceptible animals.^1^, ^38^ Furthermore, data from vaccine studies have demonstrated that high serum‐neutralizing titers are sufficient to provide protection in animal challenge models.^40^ Study of individuals exposed to RVFV occupationally has shown that naturally acquired immunity can be long‐lived, with neutralizing antibodies being readily detectable despite a lack of additional virus exposures.^41^, ^42^, ^43^ Immune correlates of protection against RVFV have been studied in mice vaccinated with an RVFV attenuated vaccine (DelNSsRVFV).^40^ Mice were protected from lethal RVFV challenge with or without depletion of CD4^+^ and/or CD8^+^ T cells, or B cells prior to RVFV challenge. However, the passive transfer of sera from immunized mice provided vaccine‐naïve mice with complete protection from lethal challenge, confirming the role of neutralizing antibodies in protection. While these studies do not preclude the role that non‐neutralizing Gn‐ and/or Gc‐specific antibodies might be playing in protection, a study by Cartwright et al. fails to demonstrate the protective capacity of three non‐neutralizing monoclonal antibodies (mAbs) compared to neutralizing mAbs.^44^ However, IgG2a versions of neutralizing mAbs conferred better protection than the corresponding IgG1 version, suggesting that antibody‐mediated effector functions may play a role in the protection.

Gn and Gc are the major antigenic determinants and targets for neutralizing antibodies arising following infection. A recent study by Wright et al. demonstrated by depletion of Gn‐ or Gc‐specific antibodies from sera of RVFV convalescent donors that the majority of the neutralizing activity is directed against Gn and a minor proportion against Gc.^39^ This is consistent with structural studies showing that Gn shields Gc in the RVFV prefusion conformation.^31^

While the immune response to RVFV infection has been the most extensively studied, antibody responses to other phlebovirus similarly reveal Gn and Gc to be the major targets for infection‐derived immunity.^45^ Similar to RVFV, Gn and Gc are targeted by neutralizing antibodies following SFTSV^46^ or TOSV infection.^47^ Convalescent sera from individuals recovered from SFTSV was shown to protect from lethal infection in a mouse model when administered after SFTSV exposure.^48^

## ANTIGENIC TARGETS ON PHLEBOVIRUSES AND SFTSV

Isolation and characterization of mAbs from convalescent donors or vaccinated animals provides a platform to characterize sites of immune vulnerability on the Gn and Gc of phleboviruses that can be targeted by mAb therapies and that can inform rational development of phlebovirus vaccines. The majority of studies isolating phlebovirus mAbs have focused on RVFV and SFTSV (summarized in Table 1). Confirming analysis of the neutralizing activity in convalescent sera, neutralizing mAbs targeting both the Gn and Gc of phleboviruses have been isolated and characterized, and their protective capacity determined. Many of the neutralizing and protective mAbs described in Table 1 represent important candidates for the development of antibody‐based therapeutics for the prevention and/or treatment of phlebovirus infections.

**TABLE 1:** Summary of phlebovirus‐specific monoclonal antibodies isolated from convalescent or vaccinated human donors or vaccinated animals.

Specificity	Domain	Origin	mAb name	Neutralizing activity	Protective capacity	Ref.
RVFV Gn	Gn domain A	Human RVFV convalescent donor	R4, R12, R13, R15, R16, R17, R19, R22	Yes	Yes	15
RVFV Gn	Gn domain A	Human RVFV convalescents or MP‐12–vaccinated humans	RVFV‐142, RVFV‐268, RVFV‐379, RVFV‐436, RVFV‐426, RVFV‐296, RVFV‐401	Yes	Yes (RVFV‐268)	50, 51
RVFV Gn	Gn domain B	Human RVFV convalescents or MP‐12–vaccinated humans	RVFV‐226, RVFV‐381, RVFV‐405, RVFV‐429,	Yes	Yes (RVFV‐226)	50
RVFV Gn	Gn domain B	Rabbits immunized with RVFV Gn	RV‐Gn1	Yes	Yes	14
RVFV Gn	Gn domain B	Macaques immunized with recombinant human adenovirus type 4 expressing RVFV Gn and Gc proteins (rHAdV4‐GnGcopt)	1332F11 1331E4	Yes	Not tested	52
SFTSV Gn	Gn domain B	Human phage library from SFTSV convalescent donor	MAb 4‐5	Yes	No	16, 53, 54
SFTSV Gn	β‐Ribbon domain	SFTSV convalescent donor	Ab10	Yes	Yes	54
TOSV Gn	Gn domain A/near transmembrane domain	TOSV convalescent donor	TVB 27, TVB 73, TVB 147, TVB 161, TVB 164	Yes	Not tested	55
RVFV Gc	Gc	Human RVFV convalescent donor	R5	Yes	No	15
RVFV Gc	Gc domain II	Human RVFV convalescents or MP‐12–vaccinated humans	RVFV‐326, RVFV‐121, RVFV‐250	Yes	Not tested	50
RVFV Gc	Fusion loop	Human RVFV convalescents or MP‐12–vaccinated humans	RVFV‐128	Yes	Not tested	50
RVFV Gc	Gc domain I	Human RVFV convalescents or MP‐12–vaccinated humans	RVFV‐249	No	Not tested	50
SFTSV Gc	Gc	SFTSV convalescent donor	No name given	Not tested	Not tested	54
RVFV Gn/Gc	Unknown quaternary epitope Gn/Gc	MP‐12–vaccinated human	RVFV‐144, RVFV‐140	Yes	Yes (RVFV‐140)	50, 51

### Neutralizing epitopes on Gn

The majority of phlebovirus‐specific neutralizing mAbs reported thus far target Gn, which is consistent with Gn being most highly exposed on the virus surface^35^ and the dominance of Gn‐specific neutralizing activity in convalescent sera.^39^ mAbs specific for RVFV Gn have been isolated from both infected and vaccinated humans, as well as from vaccinated animals.

Wang et al. isolated mAbs from an RVFV convalescent donor using antigen‐specific B cell sorting with Gn and Gc antigen baits.^15^ Eight neutralizing Gn‐specific mAbs were isolated (R4, R12, R13, R15, R16, R17, R19, and R22), all displaying low levels of somatic hypermutation and all binding to Gn domain A (Figure 2 and Table 1). A study of their mechanism of neutralization showed inhibition of RVFV interaction with Vero cells, suggesting direct inhibition of receptor binding. While all mAbs bound Gn domain A, structural analysis and epitope mapping revealed three overlapping neutralization hotspots within this domain. Sequence analysis of 108 RVFV strains showed strong conservation of residues within the mAb epitopes, predicting broad activity.

Chapman et al. selected neutralizing mAbs from both RVFV convalescent donors as well as individuals immunized with RVF MP‐12 (a live attenuated RVFV vaccine^49^) through the generation of B‐cell lymphoblastoid cell lines.^50^ In addition to Gn domain A–specific mAbs (RVFV‐142, RVFV‐268, RVFV‐379, RVFV‐436, RVFV‐426, RVFV‐296, and RVFV‐401), domain B–specific neutralizing mAbs (RVFV‐226, RVFV‐381, RVFV‐405, and RVFV‐429) were also isolated (Figure 2 and Table 1). In contrast to the neutralization mechanism of receptor binding inhibition proposed by Wang et al.,^15^ Gn‐specific mAbs that could inhibit the Gn interaction with cells were unable to completely inhibit binding of the receptor, LRP1, in biolayer interferometry competition experiments, indicating that complete blocking of the Gn–LRP1 interaction is not essential for potent neutralization by this class of mAbs.^51^ Furthermore, some Gn‐reactive mAbs isolated by Chapman et al. were shown to inhibit fusion through an indirect mode whereby mAb binding to Gn prevented subsequent exposure of the viral fusion loop.^50^ Although competition ELISAs revealed a single overlapping epitope on domain A and domain B, alanine mutagenesis revealed that the binding of mAbs was dependent on different sets of amino acids on either domain A or domain B. Domain A–specific mAbs relied heavily on amino acids present in two Gn pockets (residues 164–186 and 270–294), whereas domain B–specific mAbs were more diverse in their recognition, with some mAbs reliant on domain A for binding as well.

The immunodominance of Gn domain B was further exemplified by the isolation of RVFV domain B–reactive mAbs from rabbits immunized with recombinant RVFV Gn (mAb RV‐Gn1)^14^ and from macaques immunized with recombinant human adenovirus type 4 expressing RVFV Gn and Gc proteins (rHAdV4‐GnGcopt) (mAbs 1332F11 and 1331E4),^52^ and by isolation of a SFTSV domain B–reactive mAb (MAb 4–5) from a convalescent donor.^16^, ^53^ Despite RV‐Gn1 and MAb 4–5 showing distinct modes of binding and contacting antigenically distinct surfaces, a comparison of the Fab crystal structures in complex with RVFV Gn and SFTSV Gn, respectively, showed their epitopes localize to the same domain. Combined, these mAbs highlight domain B as a common site of immune vulnerability on phlebovirus Gn and, therefore, an important domain for rational vaccine design strategies.^14^ However, while RVFV mAb RV‐Gn1 was shown to be neutralizing and protective in a pre‐exposure RVFV infection mouse model,^14^ SFTSV MAb 4–5, which was also neutralizing *in vitro*, was not protective in an SFTSV pre‐exposure lethal challenge study in mice,^54^ suggesting different mechanisms of action for domain B–specific mAbs against specific phleboviruses and SFTSV.

No RVFV mAbs have been reported to bind the β‐ribbon domain.^15^, ^50^ However, the epitope of mAb Ab10 isolated from an SFTSV convalescent donor was shown using crosslinking coupled mass spectrometry and alanine scanning mutagenesis to be located to the Gn β‐ribbon domain and the stem region of Gn.^54^ Although further structural analysis is required to determine the exact molecular interactions, the proposed neutralization mechanism is inhibition of the un‐shielding of the Gc fusion loop. mAb Ab10 was shown to protect both pre‐ and post‐exposure from a lethal SFTSV challenge dose in a type 1 interferon‐deficient mouse model.^54^

Neutralizing mAbs (TVB 27, TVB 73, TVB 147, TVB 161, and TVB 164) have been isolated from a TOSV convalescent donor through the immortalization of memory B cells (Table 1).^55^ The neutralizing epitopes were mapped using pepscan, which revealed epitopes located in the N‐terminus (domain A) and near to the transmembrane region. Further analysis is required to determine the epitope location in TOSV Gn.

### Neutralizing epitopes on Gc

In the prefusion state, the Gc fusion loop is shielded by Gn and, therefore, this virally encoded antigen is less exposed to the immune system during infection. Indeed, only a handful of Gc‐specific mAbs have been reported thus far (Table 1). RVFV Gc‐specific mAb R5 was isolated from an RVFV convalescent donor and was found to be less potently neutralizing than Gn‐specific mAbs isolated from the same donor.^15^ mAb R5 did not protect mice from lethal challenge in either a pre‐ or post‐exposure infection model. In a study by Chapman et al., they isolated^50^ five RVFV Gc‐specific mAbs that formed three binding competition groups. Although these mAbs demonstrated neutralizing activity, some residual infection was seen in the *in vitro* assay. A mutagenesis screening approach showed three of the mAbs (belonging to Group B: RVFV‐326, RVFV‐121, and RVFV‐250) lost binding with mutations in the Gc domain II region adjacent to the fusion loop; a further mAb (belonging to Group C: RVFV‐128) lost binding with mutations in the fusion loop, and a final mAb (belonging to Group D: RVFV‐249) lost binding with mutations in domain I of Gc. The protective capacity of these Gc‐specific mAbs was not determined. Five SFTSV Gc‐reactive scFvs were isolated from a phage library generated from an SFTSV convalescent donor but the neutralization capacity or analysis of their epitopes has not been reported.^54^

### Quaternary epitopes spanning Gn–Gc

Neutralizing antibodies binding a quaternary epitope spanning multiple glycoproteins have been identified for HIV‐1.^56^, ^57^ Isolation of phlebovirus‐neutralizing mAbs targeting quaternary epitopes spanning Gn and Gc would require antigen baits displaying the native Gn/Gc configuration, which are currently not available in recombinant form. In an alternative approach, Chapman et al. selected B cells through screening for neutralization activity in the supernatants of immortalized B cells from an RVF MP‐12–vaccinated individual (a live attenuated vaccine). Two mAbs (RVFV‐140 and RVFV‐144) were able to bind cells transfected with the full M segment but not to cells expressing Gn or Gc alone, indicating binding to a quaternary Gn/Gc epitope (Table 1).^50^ While the epitope targeting by these mAbs still needs to be defined at the molecular level, a representative mAb from this group was shown to protect in an RVFV pre‐ and post‐exposure mouse model.^50^

## PROSPECTS FOR DEVELOPMENT OF CROSS‐REACTIVE NEUTRALIZING ANTIBODIES

Although mAbs have been identified that show potent neutralization and protection against RVFV or SFTSV, isolation of mAbs with cross‐neutralizing or cross‐protective activity against multiple phleboviruses has thus far proved elusive. This is despite the epitopes of RVFV‐ and STSFV‐specific mAbs being mapped to similar domains on Gn.^14^, ^16^, ^53^ Gn, which a high proportion of mAbs target, is the most exposed glycoprotein on the virus surface and as a predominant target for neutralizing antibodies elicited following infection is under the highest level of antigenic selection. Analysis of the absolute rate of nucleotide substitution ratios (dN/dS) for RVFV gene sequences sampled between 1951 and 2010 revealed regions of Gn and Gc to be under the greatest selective pressure.^14^ When comparing Gn and Gc, Gn was found to have a slightly higher mean dN/dS ratio compared to Gc. However, when Gn was separated into domains, it was found that domain B had the highest dN/dS ratio compared to domain A and the β‐ribbon domain, indicative of domain B being subject to the greatest level of antibody selective pressure and thus a dominant target of neutralizing antibodies arising from infection. However, the level of variation in this highly exposed domain will unlikely generate cross‐protective neutralizing antibodies.

In contrast, Gc is more conserved due to the shared mechanism of membrane fusion across phleboviruses,^58^ and the reduced accessibility to neutralizing antibodies^39^ leads to lower levels of antigenic selection sequence diversity.^14^, ^39^ Therefore, cross‐neutralizing epitopes present across multiple phlebovirus species will more likely be identified on Gc.

## PHLEBOVIRUS VACCINE DEVELOPMENT

The development of effective vaccines that protect animals and humans from known and newly emerging phleboviruses would have a significant impact on global health as well as the economic burden of animal deaths. As outbreaks of RVFV often precede outbreaks in humans by a few weeks,^22^ it is important to develop vaccines that can be used in animal reservoirs as well as humans. A variety of vaccine approaches against RVFV have been explored,^59^, ^60^ of which several have been evaluated in humans with the aim of generating a neutralizing antibody response. The RVFV‐inactivated vaccine TSI‐GSD‐200, although having a good safety profile in humans, ∼10% of participants immunized with it did not seroconvert despite the administration of three vaccine doses.^61^ An RVFV live attenuated vaccine, RVF MP‐12, was also safe and effective at generating neutralizing antibodies.^23^, ^24^ The fact that neutralizing RVFV mAbs from MP‐12–immunized humans have been isolated supports further development of this vaccine.^50^ Most recently, a nonreplicating simian adenovirus vectored RVFV vaccine encoding Gn and Gc (ChAdOx1 RVF) has been tested in Phase 1 trials.^25^ Similar to studies conducted in livestock,^62^, ^63^ high titers of RVFV‐neutralizing antibodies were observed in 12 of 15 participants following a single vaccination and these levels were maintained during the 3‐month follow‐up period. Interestingly, higher titers of Gc‐reactive IgG were detected than Gn‐reactive IgG. ChAdOx1 RVF is a promising vaccine candidate due to the development of a scalable manufacturing process used for the ChAdOx1 nCoV‐19 vaccine that has been used in over 180 countries.^64^, ^65^

While Phase 1 trials in humans have not yet been conducted for other phleboviruses, several approaches for SFTSV have been explored in animal models with varying degrees of success.^66^ A DNA vaccine encoding full‐length Gn, Gc, N, NS, and RdRp genes of SFTSV generated both neutralizing antibodies and a T cell response that was able to protect from lethal challenge in a ferret challenge model.^67^ A replication‐deficient human adenovirus type 5 (rAd5) expressing SFTSV Gn prime and recombinant Gn protein boost induced a potent neutralizing antibody response in mice and nonhuman primates and provided protection against lethal SFTSV challenge in mice.^68^ Furthermore, a live attenuated virus vaccine consisting of a replication‐competent rVSV vector expressing SFTSV Gn/Gc was shown to elicit broad neutralizing antibodies against both SFTSV and HRTV in both immunocompetent and immunocompromised mice and provided protection from lethal challenge.^69^ Therefore, there are several promising vaccine candidates for specific phleboviruses that may be adapted to the development of vaccines against other pathogenic and newly emerging phleboviruses.

## CONCLUSIONS AND OUTSTANDING QUESTIONS

Neutralizing antibodies play an important role in virus clearance upon infection and are able to prevent phlebovirus infection in animal challenge models.^1^, ^38^, ^40^ While neutralizing epitopes have been identified on the surface glycoproteins of RVFV, SFTSV, and TOSV, mAbs with cross‐binding or cross‐neutralizing activity against multiple phlebovirus families have not been identified. Encouragingly, a number of vaccine approaches, including recombinant Gn,^14^ live attenuated vaccines,^23^, ^24^ and viral‐vectored vaccines,^25^ readily generate high titers of Gn‐ and Gc‐specific IgG that, unlike HIV‐1 broadly neutralizing antibodies,^70^ do not require extensive somatic hypermutation for neutralization activity. Therefore, knowledge of the M segment sequence of a newly emerging phlebovirus species with pandemic potential would rapidly pave the way to vaccine development using the strategies outlined above. However, the ultimate goal in controlling phlebovirus infections is the development of vaccines that provide broad protection against both known phleboviruses that are causing ongoing zoonosis events, as well as newly emerging phleboviruses with unknown pandemic potential. Through a more complete knowledge of the sites of immune vulnerability on the Gn and Gc surface glycoproteins, conserved antigenic regions can be identified that can be targeted through reverse vaccinology and rational vaccine design. From a global perspective, the challenge remains to identify cost‐effective vaccine formulations for use in animal populations, which will be critical for reducing the phlebovirus animal reservoir and the subsequent spill‐over into human populations.

## COMPETING INTERESTS

The author reports no competing interests.

## References

[1] Daubney, R. , Hudson, J. R. , & Garnham, P. C. (1931). Enzootic hepatitis or Rift Valley Fever. An undescribed virus disease of sheep cattle and man from East Africa. Journal of Pathology, 34, 545–579.

[2] Balkhy, H. H. , & Memish, Z. A. (2003). Rift Valley fever: An uninvited zoonosis in the Arabian Peninsula. International Journal of Antimicrobial Agents, 21, 153–157. 12615379 10.1016/s0924-8579(02)00295-9

[3] Al‐Hazmi, M. , Ayoola, E. A. , Abdurahman, M. , Banzal, S. , Ashraf, J. , El‐Bushra, A. , Hazmi, A. , Abdullah, M. , Abbo, H. , Elamin, A. , Al‐Sammani, E.‐T. , Gadour, M. , Menon, C. , Hamza, M. , Rahim, I. , Hafez, M. , Jambavalikar, M. , Arishi, H. , & Aqeel, A. (2003). Epidemic Rift Valley fever in Saudi Arabia: A clinical study of severe illness in humans. Clinical Infectious Diseases, 36, 245–252. 12539063 10.1086/345671

[4] Sow, A. , Faye, O. , Ba, Y. , Ba, H. , Diallo, D. , Faye, O. , Loucoubar, C. , Boushab, M. , Barry, Y. , Diallo, M. , & Sall, A. A. (2014). Rift Valley fever outbreak, southern Mauritania (2012). Emerging Infectious Diseases, 20, 296–299. 24447334 10.3201/eid2002.131000 PMC3901467

[5] Nanyingi, M. O. , Munyua, P. , Kiama, S. G. , Muchemi, G. M. , Thumbi, S. M. , Bitek, A. O. , Bett, B. , Muriithi, R. M. , & Njenga, M. K. (2015). A systematic review of Rift Valley fever epidemiology 1931–2014. Infection Ecology & Epidemiology, 5, 28024. 26234531 10.3402/iee.v5.28024 PMC4522434

[6] World Health Organization . (2018). Rift Valley fever. WHO.

[7] Adams, M. J. , Lefkowitz, E. J. , King, A. M. Q. , Harrach, B. , Harrison, R. L. , Knowles, N. J. , Kropinski, A. M. , Krupovic, M. , Kuhn, J. H. , Mushegian, A. R. , Nibert, M. , Sabanadzovic, S. , Sanfaçon, H. , Siddell, S. G. , Simmonds, P. , Varsani, A. , Zerbini, F. M. , Gorbalenya, A. E. , & Davison, A. J. (2017). Changes to taxonomy and the International Code of Virus Classification and Nomenclature ratified by the International Committee on Taxonomy of Viruses (2017). Archives of Virology, 162, 2505–2538. 28434098 10.1007/s00705-017-3358-5

[8] Verani, P. , Nicoletti, L. , & Ciufolini, M. G. (1984). Antigenic and biological characterization of Toscana virus, a new Phlebotomus fever group virus isolated in Italy. Acta Virologica, 28, 39–47. 6143496

[9] Valassina, M. , Valentini, M. , Pugliese, A. , Valensin, P. E. , & Cusi, M. G. (2003). Serological survey of Toscana virus infections in a high‐risk population in Italy. Clinical and Diagnostic Laboratory Immunology, 10, 483–484. 12738655 10.1128/CDLI.10.3.483-484.2003 PMC154978

[10] Savage, H. M. , Godsey, M. S. , Lambert, A. , Panella, N. A. , Burkhalter, K. L. , Harmon, J. R. , Lash, R. R. , Ashley, D. C. , & Nicholson, W. L. (2013). First detection of heartland virus (Bunyaviridae: Phlebovirus) from field collected arthropods. American Journal of Tropical Medicine and Hygiene, 89, 445–452. 23878186 10.4269/ajtmh.13-0209 PMC3771279

[11] Mcmullan, L. K. , Folk, S. M. , Kelly, A. J. , Macneil, A. , Goldsmith, C. S. , Metcalfe, M. G. , Batten, B. C. , Albariño, C. G. , Zaki, S. R. , Rollin, P. E. , Nicholson, W. L. , & Nichol, S. T. (2012). A new phlebovirus associated with severe febrile illness in Missouri. New England Journal of Medicine, 367, 834–841. 22931317 10.1056/NEJMoa1203378

[12] Yu, X.‐J. , Liang, M.‐F. , Zhang, S.‐Y. , Liu, Y. , Li, J.‐D. , Sun, Y.‐L. , Zhang, L. , Zhang, Q.‐F. , Popov, V. L. , Li, C. , Qu, J. , Li, Q. , Zhang, Y.‐P. , Hai, R. , Wu, W. , Wang, Q. , Zhan, F.‐X. , Wang, X.‐J. , Kan, B. , … Li, D.‐X. (2011). Fever with thrombocytopenia associated with a novel bunyavirus in China. New England Journal of Medicine, 364, 1523–1532. 21410387 10.1056/NEJMoa1010095 PMC3113718

[13] ICTV . (2020). ICTV taxonomy history: SFTS virus . https://ictv.global/taxonomy/taxondetails?taxnode_id=20141803&src=NCBI&ictv_id=20141803

[14] Allen, E. R. , Krumm, S. A. , Raghwani, J. , Halldorsson, S. , Elliott, A. , Graham, V. A. , Koudriakova, E. , Harlos, K. , Wright, D. , Warimwe, G. M. , Brennan, B. , Huiskonen, J. T. , Dowall, S. D. , Elliott, R. M. , Pybus, O. G. , Burton, D. R. , Hewson, R. , Doores, K. J. , & Bowden, T. A. (2018). A protective monoclonal antibody targets a site of vulnerability on the surface of Rift Valley fever virus. Cell Reports, 25, 3750–3758.e4. 30590046 10.1016/j.celrep.2018.12.001 PMC6315105

[15] Wang, Q. , Ma, T. , Wu, Y. , Chen, Z. , Zeng, H. , Tong, Z. , Gao, F. , Qi, J. , Zhao, Z. , Chai, Y. , Yang, H. , Wong, G. , Bi, Y. , Wu, L. , Shi, R. , Yang, M. , Song, J. , Jiang, H. , An, Z. , … Yan, J. (2019). Neutralization mechanism of human monoclonal antibodies against Rift Valley fever virus. Nature Microbiology, 4, 1231–1241. 10.1038/s41564-019-0411-z 30936489

[16] Wu, Y. , Zhu, Y. , Gao, F. , Jiao, Y. , Oladejo, B. O. , Chai, Y. , Bi, Y. , Lu, S. , Dong, M. , Zhang, C. , Huang, G. , Wong, G. , Li, N. , Zhang, Y. , Li, Y. , Feng, W.‐H. , Shi, Y. , Liang, M. , Zhang, R. , … Gao, G. F. (2017). Structures of phlebovirus glycoprotein Gn and identification of a neutralizing antibody epitope. Proceedings of the National Academy of Sciences of the United States of America, 114, E7564–E7573. 28827346 10.1073/pnas.1705176114 PMC5594662

[17] Bao, C. J. , Guo, X. L. , Qi, X. , Hu, J. L. , Zhou, M. H. , Varma, J. K. , Cui, L. B. , Yang, H. T. , Jiao, Y. J. , Klena, J. D. , Li, L. X. , Tao, W. Y. , Li, X. , Chen, Y. , Zhu, Z. , Xu, K. , Shen, A. H. , Wu, T. , Peng, H. Y. , … Wang, H. (2011). A family cluster of infections by a newly recognized bunyavirus in eastern China, 2007: Further evidence of person‐to‐person transmission. Clinical Infectious Diseases, 53, 1208–1214. 22028437 10.1093/cid/cir732

[18] Liu, Y. , Li, Q. , Hu, W. , Wu, J. , Wang, Y. , Mei, L. , Walker, D. H. , Ren, J. , Wang, Y. , & Yu, X.‐J. (2012). Person‐to‐person transmission of severe fever with thrombocytopenia syndrome virus. Vector Borne and Zoonotic Diseases, 12, 156–160. 21955213 10.1089/vbz.2011.0758

[19] Gai, Z. , Liang, M. , Zhang, Y. , Zhang, S. , Jin, C. , Wang, S. W. , Sun, L. , Zhou, N. , Zhang, Q. , Sun, Y. , Ding, S. J. , Li, C. , Gu, W. , Zhang, F. , Wang, Y. , Bian, P. , Li, X. , Wang, Z. , Song, X. , … Li, D. (2012). Person‐to‐person transmission of severe fever with thrombocytopenia syndrome bunyavirus through blood contact. Clinical Infectious Diseases, 54, 249–252. 22095565 10.1093/cid/cir776 PMC3245727

[20] Niu, G. , Li, J. , Liang, M. , Jiang, X. , Jiang, M. , Yin, H. , Wang, Z. , Li, C. , Zhang, Q. , Jin, C. , Wang, X. , Ding, S. , Xing, Z. , Wang, S. , Bi, Z. , & Li, D. (2013). Severe fever with thrombocytopenia syndrome virus among domesticated animals, China. Emerging Infectious Diseases, 19, 756–763. 23648209 10.3201/eid1905.120245 PMC3647489

[21] Zhang, Y.‐Z. , & Xu, J. (2016). The emergence and cross species transmission of newly discovered tick‐borne Bunyavirus in China. Current Opinion in Virology, 16, 126–131. 26949898 10.1016/j.coviro.2016.02.006

[22] Hassan, A. , Muturi, M. , Mwatondo, A. , Omolo, J. , Bett, B. , Gikundi, S. , Konongoi, L. , Ofula, V. , Makayotto, L. , Kasiti, J. , Oele, E. , Onyango, C. , Gura, Z. , Njenga, K. , & Munyua, P. (2020). Epidemiological investigation of a Rift Valley fever outbreak in humans and livestock in Kenya (2018). American Journal of Tropical Medicine and Hygiene, 103, 1649–1655. 32748778 10.4269/ajtmh.20-0387 PMC7543801

[23] Pittman, P. R. , Mcclain, D. , Quinn, X. , Coonan, K. M. , Mangiafico, J. , Makuch, R. S. , Morrill, J. , & Peters, C. J. (2016). Safety and immunogenicity of a mutagenized, live attenuated Rift Valley fever vaccine, MP‐12, in a Phase 1 dose escalation and route comparison study in humans. Vaccine, 34, 424–429. 26718688 10.1016/j.vaccine.2015.12.030

[24] Pittman, P. R. , Norris, S. L. , Brown, E. S. , Ranadive, M. V. , Schibly, B. A. , Bettinger, G. E. , Lokugamage, N. , Korman, L. , Morrill, J. C. , & Peters, C. J. (2016). Rift Valley fever MP‐12 vaccine Phase 2 clinical trial: Safety, immunogenicity, and genetic characterization of virus isolates. Vaccine, 34, 523–530. 26706271 10.1016/j.vaccine.2015.11.078 PMC4731098

[25] Jenkin, D. , Wright, D. , Folegatti, P. M. , Platt, A. , Poulton, I. , Lawrie, A. , Tran, N. , Boyd, A. , Turner, C. , Gitonga, J. N. , Karanja, H. K. , Mugo, D. , Ewer, K. J. , Bowden, T. A. , Gilbert, S. C. , Charleston, B. , Kaleebu, P. , Hill, A. V. S. , & Warimwe, G. M. (2023). Safety and immunogenicity of a ChAdOx1 vaccine against Rift Valley fever in UK adults: An open‐label, non‐randomised, first‐in‐human phase 1 clinical trial. Lancet Infectious Diseases, 23, 956–964. 37060917 10.1016/S1473-3099(23)00068-3 PMC7614834

[26] Suemori, K. , Saijo, M. , Yamanaka, A. , Himeji, D. , Kawamura, M. , Haku, T. , Hidaka, M. , Kamikokuryo, C. , Kakihana, Y. , Azuma, T. , Takenaka, K. , Takahashi, T. , Furumoto, A. , Ishimaru, T. , Ishida, M. , Kaneko, M. , Kadowaki, N. , Ikeda, K. , Sakabe, S. , … Yasukawa, M. (2021). A multicenter non‐randomized, uncontrolled single arm trial for evaluation of the efficacy and the safety of the treatment with favipiravir for patients with severe fever with thrombocytopenia syndrome. PLOS Neglected Tropical Diseases, 15, e0009103. 33617533 10.1371/journal.pntd.0009103 PMC7899362

[27] Tani, H. , Komeno, T. , Fukuma, A. , Fukushi, S. , Taniguchi, S. , Shimojima, M. , Uda, A. , Morikawa, S. , Nakajima, N. , Furuta, Y. , & Saijo, M. (2018). Therapeutic effects of favipiravir against severe fever with thrombocytopenia syndrome virus infection in a lethal mouse model: Dose‐efficacy studies upon oral administration. PLoS ONE, 13, e0206416. 30365543 10.1371/journal.pone.0206416 PMC6203377

[28] Sherman, M. B. , Freiberg, A. N. , Holbrook, M. R. , & Watowich, S. J. (2009). Single‐particle cryo‐electron microscopy of Rift Valley fever virus. Virology, 387, 11–15. 19304307 10.1016/j.virol.2009.02.038 PMC2673237

[29] Freiberg, A. N. , Sherman, M. B. , Morais, M. C. , Holbrook, M. R. , & Watowich, S. J. (2008). Three‐dimensional organization of Rift Valley fever virus revealed by cryoelectron tomography. Journal of Virology, 82, 10341–10348. 18715915 10.1128/JVI.01191-08 PMC2573222

[30] Huiskonen, J. T. , ÖVerby, A. K. , Weber, F. , & GrüNewald, K. (2009). Electron cryo‐microscopy and single‐particle averaging of Rift Valley fever virus: Evidence for GN‐GC glycoprotein heterodimers. Journal of Virology, 83, 3762–3769. 19193794 10.1128/JVI.02483-08 PMC2663282

[31] Halldorsson, S. , Li, S. , Li, M. , Harlos, K. , Bowden, T. A. , & Huiskonen, J. T. (2018). Shielding and activation of a viral membrane fusion protein. Nature Communications, 9, 349. 10.1038/s41467-017-02789-2 PMC5783950 29367607

[32] Lozach, P.‐Y. , Kühbacher, A. , Meier, R. , Mancini, R. , Bitto, D. , Bouloy, M. , & Helenius, A. (2011). DC‐SIGN as a receptor for phleboviruses. Cell Host & Microbe, 10, 75–88. 21767814 10.1016/j.chom.2011.06.007

[33] Harmon, B. , Schudel, B. R. , Maar, D. , Kozina, C. , Ikegami, T. , Tseng, C.‐T. K. , & Negrete, O. A. (2012). Rift Valley fever virus strain MP‐12 enters mammalian host cells via caveola‐mediated endocytosis. Journal of Virology, 86, 12954–12970. 22993156 10.1128/JVI.02242-12 PMC3497621

[34] Dessau, M. , & Modis, Y. (2013). Crystal structure of glycoprotein C from Rift Valley fever virus. Proceedings of the National Academy of Sciences of the United States of America, 110, 1696–1701. 23319635 10.1073/pnas.1217780110 PMC3562824

[35] Halldorsson, S. , Behrens, A.‐J. , Harlos, K. , Huiskonen, J. T. , Elliott, R. M. , Crispin, M. , Brennan, B. , & Bowden, T. A. (2016). Structure of a phleboviral envelope glycoprotein reveals a consolidated model of membrane fusion. Proceedings of the National Academy of Sciences of the United States of America, 113, 7154–7159. 27325770 10.1073/pnas.1603827113 PMC4932967

[36] Zhu, Y. , Wu, Y. , Chai, Y. , Qi, J. , Peng, R. , Feng, W. H. , & Gao, G. F. (2018). The postfusion structure of the heartland virus Gc glycoprotein supports taxonomic separation of the Bunyaviral families Phenuiviridae and Hantaviridae. Journal of Virology, 92, e01558‐17. 29070692 10.1128/JVI.01558-17 PMC5730780

[37] Hulswit, R. J. G. , Paesen, G. C. , Bowden, T. A. , & Shi, X. (2021). Recent advances in Bunyavirus glycoprotein research: Precursor processing, receptor binding and structure. Viruses, 13, 353. 10.3390/v13020353 PMC7926653 33672327

[38] Peters, C. J. , Jones, D. , Trotter, R. , Donaldson, J. , White, J. , Stephen, E. , & Slone, T. W. (1988). Experimental Rift Valley fever in rhesus macaques. Archives of Virology, 99, 31–44. 3355374 10.1007/BF01311021

[39] Wright, D. , Allen, E. R. , Clark, M. H. A. , Gitonga, J. N. , Karanja, H. K. , Hulswit, R. J. G. , Taylor, I. , Biswas, S. , Marshall, J. , Mwololo, D. , Muriuki, J. , Bett, B. , Bowden, T. A. , & Warimwe, G. M. (2020). Naturally acquired Rift Valley fever virus neutralizing antibodies predominantly target the Gn glycoprotein. Iscience, 23, 101669. 33134899 10.1016/j.isci.2020.101669 PMC7588868

[40] Doyle, J. D. , Barbeau, D. J. , Cartwright, H. N. , & Mcelroy, A. K. (2022). Immune correlates of protection following Rift Valley fever virus vaccination. NPJ Vaccines, 7, 129. 36307416 10.1038/s41541-022-00551-4 PMC9616434

[41] Brown, R. D. , Scott, G. R. , & Dalling, T. (1957). Persistence of antibodies to Rift Valley fever in man. Lancet, 270, 345.

[42] Smithburn, K. C. , Mahaffy, A. F. , Haddow, A. J. , Kitchen, S. F. , & Smith, J. F. (1949). Rift Valley fever; Accidental infections among laboratory workers. Journal of Immunology, 62, 213–227. 18153372

[43] Sabin, A. B. , & Blumberg, R. W. (1947). Human infection with Rift Valley fever virus and immunity twelve years after single attack. Proceedings of the Society for Experimental Biology and Medicine, 64, 385–389. 20239431 10.3181/00379727-64-15803

[44] Cartwright, H. N. , Barbeau, D. J. , & Mcelroy, A. K. (2021). Isotype‐specific Fc effector functions enhance antibody‐mediated Rift Valley fever virus protection in vivo. mSphere, 6, e0055621. 34494884 10.1128/mSphere.00556-21 PMC8550229

[45] Serris, A. (2021). The input of structural vaccinology in the search for vaccines against Bunyaviruses. Viruses, 13, 1766. 34578349 10.3390/v13091766 PMC8473429

[46] Hofmann, H. , Li, X. , Zhang, X. , Liu, W. , Kühl, A. , Kaup, F. , Soldan, S. S. , González‐Scarano, F. , Weber, F. , He, Y. , & Pöhlmann, S. (2013). Severe fever with thrombocytopenia virus glycoproteins are targeted by neutralizing antibodies and can use DC‐SIGN as a receptor for pH‐dependent entry into human and animal cell lines. Journal of Virology, 87, 4384–4394. 23388721 10.1128/JVI.02628-12 PMC3624395

[47] Di Bonito, P. , Bosco, S. , Mochi, S. , Accardi, L. , Ciufolini, M. G. , Nicoletti, L. , & Giorgi, C. (2002). Human antibody response to Toscana virus glycoproteins expressed by recombinant baculovirus. Journal of Medical Virology, 68, 615–619. 12376972 10.1002/jmv.10227

[48] Shimada, S. , Posadas‐Herrera, G. , Aoki, K. , Morita, K. , & Hayasaka, D. (2015). Therapeutic effect of post‐exposure treatment with antiserum on severe fever with thrombocytopenia syndrome (SFTS) in a mouse model of SFTS virus infection. Virology, 482, 19–27. 25817401 10.1016/j.virol.2015.03.010 PMC7125729

[49] Ikegami, T. , Hill, T. E. , Smith, J. K. , Zhang, L. , Juelich, T. L. , Gong, B. , Slack, O. A. L. , Ly, H. J. , Lokugamage, N. , & Freiberg, A. N. (2015). Rift Valley fever virus MP‐12 vaccine is fully attenuated by a combination of partial attenuations in the S, M, and L segments. Journal of Virology, 89, 7262–7276. 25948740 10.1128/JVI.00135-15 PMC4473576

[50] Chapman, N. S. , Zhao, H. , Kose, N. , Westover, J. B. , Kalveram, B. , Bombardi, R. , Rodriguez, J. , Sutton, R. , Genualdi, J. , LaBeaud, A. D. , Mutuku, F. M. , Pittman, P. R. , Freiberg, A. N. , Gowen, B. B. , Fremont, D. H. , & Crowe, J. E. (2021). Potent neutralization of Rift Valley fever virus by human monoclonal antibodies through fusion inhibition. Proceedings of the National Academy of Sciences of the United States of America, 118, e2025642118. 33782133 10.1073/pnas.2025642118 PMC8040655

[51] Chapman, N. S. , Hulswit, R. J. G. , Westover, J. L. B. , Stass, R. , Paesen, G. C. , Binshtein, E. , Reidy, J. X. , Engdahl, T. B. , Handal, L. S. , Flores, A. , Gowen, B. B. , Bowden, T. A. , & Crowe, J. E. (2023). Multifunctional human monoclonal antibody combination mediates protection against Rift Valley fever virus at low doses. Nature Communications, 14, 5650. 10.1038/s41467-023-41171-3 PMC10499838 37704627

[52] Hao, M. , Zhang, G. , Zhang, S. , Chen, Z. , Chi, X. , Dong, Y. , Fan, P. , Liu, Y. , Chen, Y. , Song, X. , Liu, S. , Yu, C. , Li, J. , & Xia, X. (2020). Characterization of two neutralizing antibodies against Rift Valley fever virus Gn protein. Viruses, 12, 259. 32120864 10.3390/v12030259 PMC7150882

[53] Guo, X. , Zhang, L. , Zhang, W. , Chi, Y. , Zeng, X. , Li, X. , Qi, X. , Jin, Q. , Zhang, X. , Huang, M. , Wang, H. , Chen, Y. , Bao, C. , Hu, J. , Liang, S. , Bao, L. , Wu, T. , Zhou, M. , & Jiao, Y. (2013). Human antibody neutralizes severe fever with thrombocytopenia syndrome virus, an emerging hemorrhagic fever virus. Clinical and Vaccine Immunology, 20, 1426–1432. 23863504 10.1128/CVI.00222-13 PMC3889583

[54] Kim, K. H. , Kim, J. , Ko, M. , Chun, J. Y. , Kim, H. , Kim, S. , Min, J.‐Y. , Park, W. B. , Oh, M.‐D. , & Chung, J. (2019). An anti‐Gn glycoprotein antibody from a convalescent patient potently inhibits the infection of severe fever with thrombocytopenia syndrome virus. PLoS Pathogens, 15, e1007375. 30707748 10.1371/journal.ppat.1007375 PMC6380599

[55] Gandolfo, C. , Prathyumn, S. , Terrosi, C. , Anichini, G. , Gori Savellini, G. , Corti, D. , Bracci, L. , Lanzavecchia, A. , Roman‐Sosa, G. , & Cusi, M. G. (2021). Identification of a neutralizing epitope on TOSV Gn glycoprotein. Vaccines, 9, 924. 34452049 10.3390/vaccines9080924 PMC8402642

[56] Falkowska, E. , Le, K. M. , Ramos, A. , Doores, K. J. , Lee, J. H. , Blattner, C. , Ramirez, A. , Derking, R. , Van Gils, M. J. , Liang, C.‐H. , Mcbride, R. , Von Bredow, B. , Shivatare, S. S. , Wu, C.‐Y. , Chan‐Hui, P.‐Y. , Liu, Y. , Feizi, T. , Zwick, M. B. , Koff, W. C. , … Burton, D. R. (2014). Broadly neutralizing HIV antibodies define a glycan‐dependent epitope on the prefusion conformation of gp41 on cleaved envelope trimers. Immunity, 40, 657–668. 24768347 10.1016/j.immuni.2014.04.009 PMC4070425

[57] Huang, J. , Kang, B. H. , Pancera, M. , Lee, J. H. , Tong, T. , Feng, Y. , Imamichi, H. , Georgiev, I. S. , Chuang, G.‐Y. , Druz, A. , Doria‐Rose, N. A. , Laub, L. , Sliepen, K. , Van Gils, M. J. , De La Peña, A. T. , Derking, R. , Klasse, P.‐J. , Migueles, S. A. , Bailer, R. T. , … Connors, M. (2014). Broad and potent HIV‐1 neutralization by a human antibody that binds the gp41–gp120 interface. Nature, 515, 138. 25186731 10.1038/nature13601 PMC4224615

[58] Spiegel, M. , Plegge, T. , & Pohlmann, S. (2016). The role of Phlebovirus glycoproteins in viral entry, assembly and release. Viruses, 8, 202. 27455305 10.3390/v8070202 PMC4974537

[59] Wichgers Schreur, P. J. , Bird, B. H. , Ikegami, T. , Bermúdez‐Méndez, E. , & Kortekaas, J. (2023). Perspectives of next‐generation live‐attenuated Rift Valley fever vaccines for animal and human use. Vaccines, 11, 707. 36992291 10.3390/vaccines11030707 PMC10054419

[60] Kitandwe, P. K. , McKay, P. F. , Kaleebu, P. , & Shattock, R. J. (2022). An overview of Rift Valley fever vaccine development strategies. Vaccines, 10(11), 1794. 36366303 10.3390/vaccines10111794 PMC9697312

[61] Pittman, P. R. , Liu, C. T. , Cannon, T. L. , Makuch, R. S. , Mangiafico, J. A. , Gibbs, P. H. , & Peters, C. J. (1999). Immunogenicity of an inactivated Rift Valley fever vaccine in humans: A 12‐year experience. Vaccine, 18, 181–189. 10501248 10.1016/s0264-410x(99)00218-2

[62] Stedman, A. , Wright, D. , Wichgers Schreur, P. J. , Clark, M. H. A. , Hill, A. V. S. , Gilbert, S. C. , Francis, M. J. , Van Keulen, L. , Kortekaas, J. , Charleston, B. , & Warimwe, G. M. (2019). Safety and efficacy of ChAdOx1 RVF vaccine against Rift Valley fever in pregnant sheep and goats. NPJ Vaccines, 4, 44. 31646004 10.1038/s41541-019-0138-0 PMC6802222

[63] Warimwe, G. M. , Gesharisha, J. , Carr, B. V. , Otieno, S. , Otingah, K. , Wright, D. , Charleston, B. , Okoth, E. , Elena, L.‐G. , Lorenzo, G. , Ayman, E.‐B. , Alharbi, N. K. , Al‐Dubaib, M. A. , Brun, A. , Gilbert, S. C. , Nene, V. , & Hill, A. V. S. (2016). Chimpanzee adenovirus vaccine provides multispecies protection against Rift Valley fever. Scientific Reports, 6, 20617. 26847478 10.1038/srep20617 PMC4742904

[64] Voysey, M. , Clemens, S. A. C. , Madhi, S. A. , Weckx, L. Y. , Folegatti, P. M. , Aley, P. K. , Angus, B. , Baillie, V. L. , Barnabas, S. L. , Bhorat, Q. E. , Bibi, S. , Briner, C. , Cicconi, P. , Collins, A. M. , Colin‐Jones, R. , Cutland, C. L. , Darton, T. C. , Dheda, K. , Duncan, C. J. A. , … Zuidewind, P. (2021). Safety and efficacy of the ChAdOx1 nCoV‐19 vaccine (AZD1222) against SARS‐CoV‐2: An interim analysis of four randomised controlled trials in Brazil, South Africa, and the UK. Lancet, 397, 99–111. 33306989 10.1016/S0140-6736(20)32661-1 PMC7723445

[65] Joe, C. C. D. , Jiang, J. , Linke, T. , Li, Y. , Fedosyuk, S. , Gupta, G. , Berg, A. , Segireddy, R. R. , Mainwaring, D. , Joshi, A. , Cashen, P. , Rees, B. , Chopra, N. , Nestola, P. , Humphreys, J. , Davies, S. , Smith, N. , Bruce, S. , Verbart, D. , … Douglas, A. D. (2022). Manufacturing a chimpanzee adenovirus‐vectored SARS‐CoV‐2 vaccine to meet global needs. Biotechnology and Bioengineering, 119, 48–58. 34585736 10.1002/bit.27945 PMC8653296

[66] Yoshikawa, T. (2021). Vaccine development for severe fever with thrombocytopenia syndrome. Viruses, 13, 627. 33917632 10.3390/v13040627 PMC8067456

[67] Kwak, J.‐E. , Kim, Y.‐I. , Park, S.‐J. , Yu, M.‐A. , Kwon, H.‐I. , Eo, S. , Kim, T.‐S. , Seok, J. , Choi, W.‐S. , Jeong, J. H. , Lee, H. , Cho, Y. , Kwon, J. A. , Jeong, M. , Maslow, J. N. , Kim, Y.‐E. , Jeon, H. , Kim, K. K. , Shin, E.‐C. , … Park, S.‐H. (2019). Development of a SFTSV DNA vaccine that confers complete protection against lethal infection in ferrets. Nature Communications, 10, 3836. 10.1038/s41467-019-11815-4 PMC6707330 31444366

[68] Kim, J.‐Y. , Jeon, K. , Hong, J. J. , Park, S.‐I. , Cho, H. , Park, H.‐J. , Kwak, H. W. , Park, H.‐J. , Bang, Y.‐J. , Lee, Y.‐S. , Bae, S.‐H. , Kim, S.‐H. , Hwang, K.‐A. , Jung, D.‐I. , Cho, S. H. , Seo, S. H. , Kim, G. , Oh, H. , Lee, H.‐Y. , … Nam, J.‐H. (2023). Heterologous vaccination utilizing viral vector and protein platforms confers complete protection against SFTSV. Scientific Reports, 13, 8189. 37210393 10.1038/s41598-023-35328-9 PMC10199661

[69] Dong, F. , Li, D. , Wen, D. , Li, S. , Zhao, C. , Qi, Y. , Jangra, R. K. , Wu, C. , Xia, D. , Zhang, X. , Deng, F. , Chandran, K. , Zou, Z. , Yuan, F. , & Zheng, A. (2019). Single dose of a rVSV‐based vaccine elicits complete protection against severe fever with thrombocytopenia syndrome virus. NPJ Vaccines, 4, 5. 30701094 10.1038/s41541-018-0096-y PMC6347601

[70] Burton, D. R. , & Mascola, J. R. (2015). Antibody responses to envelope glycoproteins in HIV‐1 infection. Nature Immunology, 16, 571–576. 25988889 10.1038/ni.3158 PMC4834917

